# Effects of Beetroot Juice Supplementation on a 30-s High-Intensity Inertial Cycle Ergometer Test

**DOI:** 10.3390/nu9121360

**Published:** 2017-12-15

**Authors:** Raul Domínguez, Manuel Vicente Garnacho-Castaño, Eduardo Cuenca, Pablo García-Fernández, Arturo Muñoz-González, Fernando de Jesús, María Del Carmen Lozano-Estevan, Sandro Fernandes da Silva, Pablo Veiga-Herreros, José Luis Maté-Muñoz

**Affiliations:** 1 College of Health Sciences, Alfonso X el Sabio University, 28691 Madrid, Spain; pablgafe@uax.es (P.G.-F.); amunogon@uax.es (A.M.-G.); fdejesus@uax.es (F.d.J.); mloza@uax.es (M.D.C.L.-E.); pveigher@uax.es (P.V.-H.); jmatmuo@uax.es (J.L.M.-M.); 2 GRI-AFIRS, School of Health Sciences, TecnoCampus-Pompeu Fabra University, 08005 Barcelona, Spain; mgarnacho@escs.tenocampus.cat (M.V.G.-C.); educuen@hotmail.com (E.C.); 3 Studies Research Group in Neuromuscular Responses (GEPRE N), University of Lavras, 37200-000 Lavras, Brazil; sandrofs@gmail.com

**Keywords:** beet, nitrate, physical activity, sport, supplement

## Abstract

**Background:** Beetroot juice (BJ) is rich in inorganic nitrates and has proved effective at increasing blood nitric oxide (NO) levels. When used as a supplement BJ has shown an ergogenic effect on cardiorespiratory resistance exercise modalities, yet few studies have examined its impact on high intensity efforts. **Objective:** To assess the effects of BJ intake on anaerobic performance in a Wingate test. **Methods:** Fifteen trained men (age 21.46 ± 1.72 years, height 1.78 ± 0.07 cm and weight 76.90 ± 8.67 kg) undertook a 30-s maximum intensity test on an inertial cycle ergometer after drinking 70 mL of BJ (5.6 mmol NO_3_^−^) or placebo. **Results:** Despite no impacts of BJ on the mean power recorded during the test, improvements were produced in peak power (6%) (*p* = 0.034), average power 0–15 s (6.7%) (*p* = 0.048) and final blood lactate levels (82.6%) (*p* < 0.001), and there was a trend towards a shorter time taken to attain peak power (−8.4%) (*p* = 0.055). **Conclusions:** Supplementation with BJ has an ergonomic effect on maximum power output and on average power during the first 15 s of a 30-s maximum intensity inertial cycle ergometer test.

## 1. Introduction

Beetroot juice (BJ) is a source of inorganic nitrate (NO_3_^−^) found in other vegetables or used as preservatives for processed meat products [1]. After the intake of BJ, around 25% of the NO_3_^−^ present is reduced by bacteria in the mouth to nitrite (NO_2_^−^). As it reaches the stomach, some of this NO_2_^−^ is reduced to nitric oxide (NO) [2], and subsequently absorbed along with the nonreduced nitrite in the gut passing into the bloodstream [3] where blood NO and NO_2_^−^ concentrations rise. Besides this rise in NO levels produced after consuming NO_3_^−^ [4], in situations of low oxygen levels the NO_2_^−^ present in blood may be again reduced to NO [3]. Thus, the final result of taking a BJ supplement is that blood NO levels rise. 

NO plays a key role in several physiological, hemodynamic and metabolic events [5]. NO causes blood vessel dilation through mediation by guanylate cyclase [6], increasing blood flow to the muscles and reducing VO_2_ at a given work rate [7]. Studies have indeed shown that NO has beneficial effects on muscle contraction [8] and biogenesis [9] and mitochondrial efficiency [10]. Nitric oxide plays a role in efforts that require an oxidative-type of energy metabolism as in endurance exercises performed at a work rate lower than VO_2max_ and of duration longer than 5 min [11]. In these high-intensity efforts, many studies—though not all [12,13,14,15,16]—have measured performance or endurance indicators such as economy following the intake of BJ [17,18,19,20,21,22,23,24,25,26,27,28]. Hence, in endurance exercise modalities, BJ supplementation has been reported to reduce VO_2_ at work rates equivalent to the ventilatory threshold (VT) [10], first lactate threshold (LT1), second lactate threshold (LT2) [26], 45% VO_2max_ [18], 50% VO_2max_ [25], 60% VO_2max_ in conditions of normal oxygen levels [21] and low levels [24,28], 65% VO_2max_ [18] and 70% VO_2max_ [25,28]. In addition, BJ supplementation has shown an ergogenic effect in cycle ergometry tests until exhaustion executed at work rates equivalent to 60% VO_2max_, 70% VO_2max_, 80% VO_2max_ [13], 90% VO_2max_ [25] and to 70% [20] or 75% [22] between VT and VO_2max_, as well as improved performance at 4- [29], 10- [23] and 16-km tests in normoxia [17] and hypoxia [24], 50 miles in normoxia [19] and of 30 min in hypoxia [27].

Apart from endurance efforts, other sport modalities exist in which the predominant energy metabolism, rather than involve oxidative energy processes, entails pathways that are independent of oxygen as is the case for explosive or high intensity efforts [30]. Explosive efforts are those lasting under 6 s in which the main energy metabolism pathway is the high-energy phosphagen system and there is some participation also of glycolysis and oxidative phosphorylation [31]. This pathway gradually contributes more to energy production until it accounts for 50% of this at 6 s [31]. High-intensity efforts are those of duration 6 to 60 s that feature a major contribution of glycolytic metabolism and smaller participation of high-energy phosphagens and oxidative phosphorylation [30]. Compared to endurance efforts, these high intensity efforts potentially have an even greater capacity to increase blood NO concentrations in response to BJ supplementation. This is because during the execution of this type of exercise movement, in which the main energy metabolism is independent of oxidation reactions, a drop is produced in the partial pressure of oxygen and pH in muscle and venous and capillary blood [32], and these conditions promote the reduction of NO_2_^−^ to NO [3]. 

Studies in animals have shown that NO’s blood flow improving effect is greater for type II than type I motor units [5,29]. Further, also in animals it has been noted that the power production improvement produced in response to BJ is specific to motor type II units [33]. This is because this type of muscle unit has a greater power production capacity and is designed to obtain energy via non-oxidative pathways. This could be due to the greater capacity of these units to store glycogen and muscular creatine [34], as well as proteins such as carnosine [35], which have a buffering effect at the intracellular level [36]. Thus, BJ intake could have an ergogenic effect during both explosive efforts and high intensity efforts. A 30-s maximum sprint test on a cycle ergometer (Wingate test) can be used to assess performance at high intensity efforts by determining power output and glycolytic capacity [37]. In addition, explosive efforts can be assessed in the first 5 s of the Wingate test, as in this interval adenosine triphosphate (ATP) resynthesis occurs mainly via the high-energy phosphagen system [38]. Accordingly, in the present study, we examined the effects of BJ supplementation on anaerobic performance in a Wingate test conducted by athletes trained in sports modalities with a high glycolytic energy metabolism component. 

## 2. Materials and Methods 

### 2.1. Participants

Participants were 15 male undergraduates of Physical Activity and Sport Sciences with experience with the Wingate test (they had performed at least one test in the month before the study onset). Descriptive data for the study population are provided in Table 1. Participation in the study was voluntary, though subjects were required to fulfil the following inclusion criteria: (a) more than two years’ experience in sports modalities with a high glycolytic energy metabolism component (speed tests in athletic sports and swimming, combat and team sports); (b) not considered an elite athlete; (c) an absence of cardiovascular, lung, metabolic, or neurologic disease or of an orthopaedic disorder that could limit cycle ergometry performance; (d) no medication; (e) no smoking; (f) no nutritional supplements in the six months prior to the study onset. 

The subjects recruited were asked to attend a meeting the week before the study outset. In this meeting, three investigators informed them of the study protocol and gave them instructions about diet control and resolved any concerns they had. At the end of the meeting, they all signed an informed consent form. The study protocol was approved by the Ethics Committee of the Universidad Alfonso X El Sabio, Madrid, Spain (code number 1.010.704).

### 2.2. Study Design

Participants attended two testing sessions at the Exercise Physiology lab within the same time frame (±0.5 h) 72 h apart. From 72 h before the first session until the end of the study, subjects undertook no type of physical exercise. As soon as they arrived at the laboratory, in a random and double-blind fashion, subjects were given a BJ or placebo supplement ensuring that 50% of the subjects randomly took BJ in the first session and placebo in the second or vice versa. This meant that half the subjects in each session worked under one of the two experimental conditions. Three hours after intake of the supplement, subjects started a Wingate cycle ergometer test session including a warm-up. 

### 2.3. Nutritional Intervention and Dietary Control

As the blood NO_2_^−^ peak occurs 2–3 h post-ingestion, the supplement was administered 3 h before the endurance test [11]. The use of oral antiseptics can prevent increased blood NO_2_^−^ levels after the intake of NO_3_^−^ because of their bactericidal effect on the bacteria in the mouth. Thus, participants were asked to refrain from brushing their teeth or using a mouthwash, chewing gum or sweets that could contain a bactericidal substance such as chlorhexidine or xylitol in the 24 h prior to the test sessions.

Subjects were also instructed to avoid drinks containing caffeine during these 24 h due to its ergogenic effect [39]. The intake of alcohol was also restricted the day before the study start. 

As an individual’s diet can affect energy metabolism during exercise, subjects were given guidelines to ensure that 48 h before each of the test sessions, they followed a similar diet consisting of 60% carbohydrates, 30% lipids and 10% proteins and avoiding foods with high NO_3_^−^ contents (beetroot, celery, arugula, lettuce, spinach, turnip, endives, leak, parsley, cabbage). Participants were provided with a list of vegetables they should avoid the day before the study outset. 

Each subject randomly took the supplement by drinking the contents of a randomly assigned bottle containing 70 mL of BJ concentrate Beet-It-Pro Elite Shot (Beet IT; James White Drinks Ltd., Ipswich, UK) or placebo. The placebo was prepared by dissolving 1 g of powdered BJ (ECO Saludviva, Alicante, Spain) in a litre of mineral water and adding lemon juice to imitate the taste of the commercial supplement. Although the beetroot juice present in the placebo could have a minimum content of NO_3_^−^, the small proportion of desiccated beetroot juice in each bottle of placebo (0.015 g), along with the restricted intake of foods rich in NO_3_^−^ 48 h before the start of each session ensured that subjects working under the placebo condition were depleted of NO_3_^−^. 

Both drinks (BJ and placebo) were supplied in an unlabeled, 100-mL, brown glass bottle.

All participants were warned of the possible side-effects of BJ: gastrointestinal problems and the red appearance of urine and faeces. 

### 2.4. Wingate Test

The Wingate test was started with the subject stopped. Before the test, the following instructions were given by the investigators: (i) in the first seconds of the test, they should pedal from 0 rpm to the greatest pedalling velocity possible (rpm) in the shortest time possible; and (ii) maintain this high power level during the longest time possible until the test end.

For the test, a Monark cycle ergometer (Ergomedic 828E, Vansbro, Sweden) was used. This ergometer consists of a metal wheel with a band which, through friction, offers resistance to pedalling. This resistance may be regulated as the band is connected to a pendulum that presses on it and this pressure is modified by adjusting a screw under the handle bar. Vertical elevation of the pendulum indicates the kilograms (kg) of friction exerted on the wheel. This friction of the band against the wheel is measured in kilogram force (kgf) defined as the force acting on a 1-kg mass subjected to the acceleration of gravity. 

The Monark cycle ergometer has a cog of 52 teeth and a pinion of 14 teeth, causing the conversion of 3.71 revolutions of the wheel for each complete circle of the pedals. The wheel perimeter is 1.62 m and, as for each pedal the wheel spins 3.71 times, the wheel covers 6 m for each complete pedal revolution. The revolutions per minute (rpm) are counted by a magnet system on the pedalling axel and indicated on the speedometer. To calculate the power exerted on the pedals we need to multiply force by the movement velocity:

Power = Force on the pendulum (kgf) × Pedalling velocity (rpm)

The force exerted by the band friction is read on the pendulum and expressed in kgf. Velocity is obtained multiplying the rpm of the pedals by the wheel’s revolutions. When we multiply the kgf by the metres covered per minute we get kilopondmetres (kpm). To convert kpm into watts (W) one has to divide by 6.12:

Kilopondmetres to watts = 6.12 kpm = 1 watt

As for the Monark cycle ergometer, each complete pedal circle makes the wheel advance 6 m, each rpm is equivalent to 6 m/min. Thus, using this cycle ergometer, by multiplying the rpm by the kgf indicated by the pendulum, we obtain as a result the power in watts. For data extraction, the display of the Monark cycle ergometer was recorded with a video camera where the rpm during the whole test appeared. Subsequently, the video recording was transferred to the program Kinovea (version 0.8.15, France) which reproduces 30 photo frame/s and the rpm where compiled for each second. Next, we used the equation to determine the watts generated in each second of the test. 

Subjects first performed a 5-min warm-up consisting of light cycling with the workload and cadence set by the subject followed by 1 min of rest. After this rest period, subjects executed a specific warm up of 3 min of pedalling at a rate of 60 rpm with a workload of 2 kgf and a sprint at maximum intensity in the last 5 s of each minute. After 3 min of rest, the Wingate test was started.

The test consisted of 30 s of cycling at maximum effort with a load (kgf) corresponding to 7.5% of the subject’s body weight [40]. Participants were instructed to pedal as fast as possible to reach the maximum rpm in the shortest time possible and to try to maintain this pedalling speed until the end of the test. Two of the authors motivated the subjects during the test duration. As soon as the test was completed, the subjective rate of perceived exertion (RPE) scale used to rate leg muscle, cardiorespiratory and general perceived exertion.

Just before the warm up and 3 min after the test end, an examiner took a finger prick blood sample (5 μL) from the left index finger for blood lactate determination using a Lactate ProTM 2 LT-1710 blood analyzer (Arkray Factory Inc., KDK Corporation, Shiga, Japan). 

Besides blood lactate and muscle, cardiorespiratory and general RPE, the power (W) variables obtained in the test were analyzed through their transformation of the product of rpm × kgf at W. In this way, the variables of W for each second were examined, obtaining cutoffs for 5-s, 10-s and 30-s intervals during the course of the test. Further variables recorded were: peak power (W_peak_), time-to-W_peak_, minimum W (W_min_), mean power and fatigue index ((W_peak_ − W_min_)/W_peak_ × 100). 

### 2.5. Statistical Analysis

Initially we confirmed the normal distribution of the data using the Shapiro–Wilk test. 

The Student *t*-test for related samples was used to compare the performance variables recorded for the two experimental conditions (placebo and BJ).

All data are provided as the mean (M) and standard deviation (SD). All statistical tests were performed using the software package SPSS version 19.0 (SPSS, Chicago, IL, USA).

## 3. Results

Capillary blood lactate levels recorded before (resting lactate) and after the Wingate test (final lactate) and RPE scores after the test are provided in Table 2. The only significant difference detected was an 82.6% higher final lactate level in the group of subjects who took BJ supplements (*p* < 0.05).

The power variables recorded in the Wingate test are shown in Table 3. These data revealed that despite no differences in mean power (*p* = 0.796) between the two supplementation groups, peak power was significantly higher in the BJ vs. placebo group (5.4%; *p* = 0.034) and a trend toward significance was observed (*p* = 0.055) in time-to-peak power (−8.4% for BJ vs. placebo). When comparing power variables recorded at the start and end of the test between supplementation groups, average power 0–5 s was significantly higher in BJ (9.5%; *p* =0.05), while no differences emerged in average power 25–30 s or in the fatigue index (*p* = 0.538).

**Figure 1:**
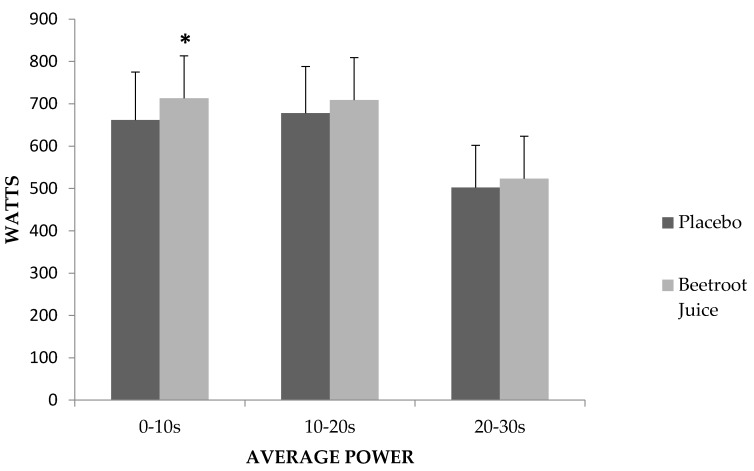
Average power recorded in the intervals 0–10, 10–20 and 20–30 s; * significant difference between beetroot juice and placebo (*p* < 0.05).

**Figure 2:**
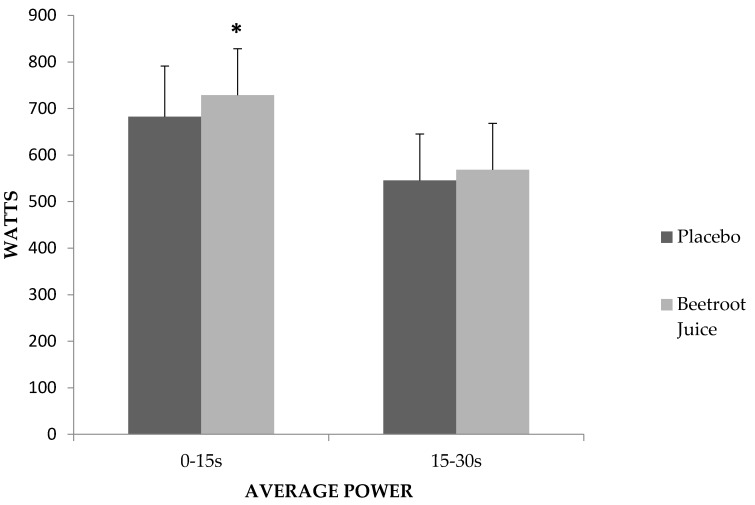
Average power recorded in the intervals 0–15 and 15–30 s; * significant difference between beetroot juice and placebo (*p* < 0.05).

When we examined average power in 10-s intervals, average power 0–10 s (placebo = 661.44 ± 113.6 W; CV: 17.2%, BJ = 713.03 ± 116.8 W; CV: 16.4%) was higher in BJ (7.8%; *p* = 0.022), but no significant differences between the conditions were produced in average power 10–20 s (placebo = 677.95 ± 110.2 W; CV: 16.3%, BJ = 708.82 ± 121.3 W; CV: 17.1%) or average power 20–30 s (*p* = 0.238 and *p* = 0.436 respectively) (placebo = 502.57 ± 99.6 W; CV: 19.8%, BJ = 523.4 ± 106.8 W; CV: 20.4%) (Figure 1). Figure 2 shows that when considering 15-s intervals, a significant difference between BJ and placebo was produced in average power 0–15 s (6.7%; *p* = 0.048) (placebo = 682.60 ± 108.9 W; CV: 16.0%, BJ = 728.59 ± 118.3 W; CV: 16.2%) but not in average power 15–30 s (*p* = 0.365) (placebo = 545.36 ± 99.9 W; CV: 18.3%, BJ = 568.24 ± 107.9 W; CV: 19.0%).

**Figure 3:**
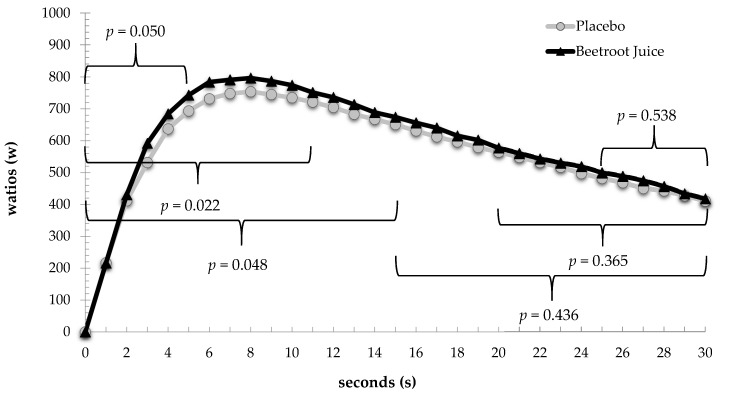
Power curves recorded during the Wingate test in the placebo and beetroot juice supplementation groups. The figure shows that during the first 15 s of the test (0–5 s, 0–10 s and 0–15 s) significant differences in power emerged between the two experimental conditions.

**Figure 4:**
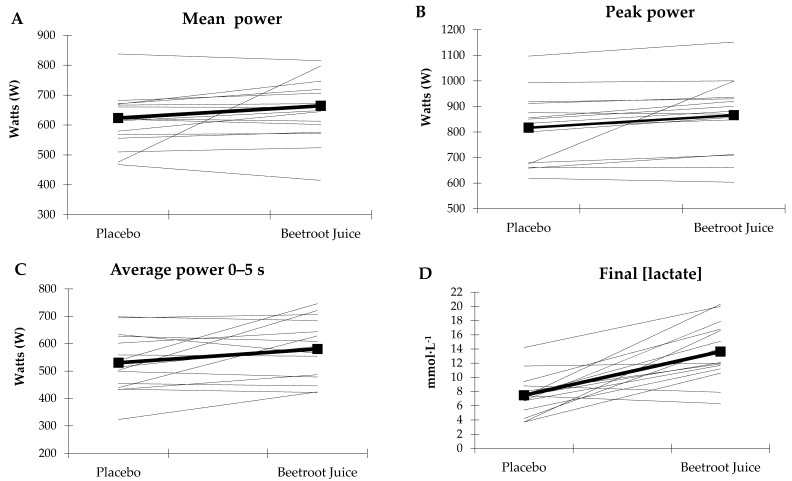
Mean power (**A**); peak power (**B**); average power 0–5 s (**C**) and Final (lactate) (**D**) recorded in all participants (dashed line) and average for the sample (continuous line).

Figure 3 visually illustrates how the W values recorded were considerably higher during the first seconds of the Wingate test and reached their greatest values in the upper part of the curve while Figure 4 show the individual and mean group response of the main variables analyzed.

## 4. Discussion

The main finding of our study was that BJ supplementation was able to significantly improve the power developed during the first 15 s of a Wingate test, with impacts on W_peak_ and a trend towards a shorter time-to-peak power (*p* = 0.055). This improved peak power production produced after the intake of BJ (6%) coincides with the results reported in studies examining its impacts on knee-extension exercises (6%) [41] and on inertial cycle ergometry (6%) [42], though the dose used in our study was 5.6 mmol NO_3_^−^ vs. 11.2 mmol NO_3_^−^ used in other investigations [41,42]. However, in this last study, BJ supplementation led to no such improvements in average and peak power during a Wingate test executed on an isokinetic cycle ergometer rather than an inertial one as in the test employed here [40].

Despite reports of significantly raised NO_2_^−^ levels in response to BJ doses of 8.4 mmol NO_3_^−^ and 16.8 mmol NO_3_^−^ compared with a dose of 4.2 mmol NO_3_^−^ [43], our finding of improved peak power attributable to the intake of 5.6 mmol NO_3_^−^ BJ before the Wingate test supports the results described by Rimer et al. [42] in response to a dose of 11.2 mmol NO_3_^−^. To explain the lack of an effect of BJ on an isokinetic Wingate test, we need to consider the characteristics of the different ergometers used in the study by Rimer’s group. As power reflects the relationship between force and velocity, for an inertial cycle ergometer in which the load remains constant (fixed at a load relative to a percentage of body weight) [44], any changes produced in power production are attributable only to modifications in pedalling cadence [45]. In contrast, when performing the test on an isokinetic cycle ergometer, the pedalling cadence is fixed and power is interpreted as the force exerted at a given velocity. Because pedalling cadence is known to correlate highly with knee and hip angular velocities, this cadence is used to indicate the shortening velocity of the muscles involved in both these joints [45]. Hence, improvements in the power produced when pedalling on inertial cycle ergometers are sensitive to changes in power output associated with velocity, while improvements when using an isokinetic cycle ergometer are related to variations in force. As one of the functions of NO is to reduce muscle shortening velocity [46], the beneficial effects of BJ supplementation may perhaps not be observed when using an isokinetic ergometer. 

Among the factors that affect the production of power, we should highlight the influence of the type of muscle motor unit recruited, as type II muscle fibres show a greater contraction velocity and force [47]. Accordingly, it has been observed that the improved peak power produced following BJ intake is specific to type II motor units [48]. Studies in animals have also shown that the effects of BJ supplementation on blood flow [5] and force production [33] are only observed in type II motor units. In effect, studies in animal models have shown that NO increases the effects of acetylcholine exclusively in type II muscle fibres [49]. An improved action of acetylcholine may enhance motor neuron depolarization [49]. Besides, BJ increases the expression of calsequestrin [29], increasing calcium release from the sarcoplasmic reticulum to the muscle fibre sarcoplasm [50]. At this site, calcium binds to tropomyosin and troponin promoting actin and myosin crossover [51]. Increased action potential succession and the presence of calcium could promote trains of action potentials thus increasing peak power output [52]. In effect, this has been observed by monitoring electromyographic activity during maximum intensity efforts [53].

In addition to the effects of BJ supplementation on force production in type II motor units, there have been reports that BJ reduces ATP demands during the exercise effort [4,54], manifesting as the reduced degradation of phosphocreatine (PCr) both in low and high intensity exercise [54]. A diminished PCr cost during the maximum intensity effort would delay the depletion of PCr reserves [6]. Given the essential role of PCr in high intensity efforts [20], its delayed depletion during the Wingate test should help maintain greater power peaks during the first part of the test, thus explaining the significant improvement noted in average power 0–15 s (6.7%). 

The effects of BJ reported here are consistent with those of a study in which the effects of supplementation with nitrate salts were examined in a Wingate test, also performed on an inertial cycle ergometer in a population of similar characteristics (CrossFit athletes). Thus, it was observed in CrossFit athletes that supplementation with 8 mmol of potassium nitrate led to a 6.6% improvement in peak power [55], comparable to the present finding of 6%. Also, while no significant improvement was observed in the average power developed during the test, a trend towards significance was noted (*p* = 0.08) [55]. However, as these authors did not compare power production across test intervals, it is not known whether a significant improvement was produced during the first 15 s of the test as noted in our study.

The effects of BJ [42] or nitrate salts [46] on maximum power produced during cycle ergometry mediated by increased NO concentrations or reduced PCr degradation rate [5,54] could also explain the findings of several studies: a greater number of repetitions (26.1 vs. 21.8) of 15-s bouts of cycle ergometry executed at 170% of maximum aerobic power (MAP) with 30-s rest periods [41]; improved power developed during 24 sets of 6-s work periods and 24-s of rest (~7%) [56]; or improved cycle ergometry work accomplished in 5 sets of 6 s followed by 14 s of rest (~3.5%) in the middle and end of a protocol consisting of 2 × 40 min that simulated the demands of a team sport’s match [23].

The increase observed here in blood lactate concentrations (82.6%) in response to BJ supplementation is similar (106.3%) to that detected in rats given an injection of NO_2_^−^ [57]. Blood lactate concentrations are considered to indicate the glycolytic contribution to energy metabolism [54], though the transfer of lactate to the bloodstream depends on the extent of capillarization and muscle perfusion. In a 30-s maximum load test such as the Wingate, in which type II motorneurons are recruited and there is a highly glycolytic metabolism, blood lactate concentrations are much lower than those of muscle lactate and several minutes are needed for blood and muscle concentrations to reach a balance [58]. It is possible that increased blood flow to type II motor units following BJ supplementation [5], could have led to increased blood lactate concentrations [59]. This possible effect of BJ [5] could be responsible for the increase in blood lactate levels observed after the high-intensity effort in our study and in the study by Glean et al. [57]. 

Another possible explanation for the increase produced in blood lactate concentrations could be elevated glycolytic activity [57]. Accordingly, the increased power produced in our study would be the consequence of a glycolytic type metabolism during the exercise effort. This mechanism could explain the ergogenic effect of this supplement detected in our study and in others [54]. Wylie et al. [56] observed increased blood lactate levels along with improved performance at a cycle ergometer protocol consisting of 24 sets of 6 s and 24-s rest periods, while Mosher et al. [58] noted an increased number of repetitions accomplished until exhaustion when lifting a load equivalent to 60% of one maximum repetition (1 RM) during bench press exercise. As the impacts on power output of BJ supplementation are attributed to the improved performance of type II motor units characterized by a greater dependence on glycolytic energy metabolism, this leads to a greater increase in blood lactate following the exercise effort [59]. In any case, neither of these two explanations are exclusive such that it could be that the increase in blood lactate produced at the end of the test were the consequence of a greater amount of work executed by type II motor units as well as their greater blood supply. 

### Limitations

Given that the effects of BJ supplementation are mediated by its capacity to raise levels of NO_2_^−^ which later may be reduced to NO, a limitation of our study was that we were unable to measure blood NO_2_^−^ levels. Moreover, since prior studies have shown a greater effect of BJ doses of 8.4 and 16.8 mmol NO_2_^−^ vs. 4.2 mmol NO_2_^−^ to increase blood NO_2_^−^ levels and improve endurance performance [43], we could have compared the impacts of the dose of 5.6 mmol NO_2_^−^ employed with that of a higher dose (11.2 mmol). The increased blood lactate concentrations observed here and in other studies [57] could either be due to a potentiating effect of BJ on blood flow which would accelerate the passage of lactate to the blood or to an increase in glycolytic activity. Hence, by monitoring blood lactate kinetics after the Wingate test, we could have examined whether these higher lactate concentrations persisted during the recovery period (due to increased glycolytic activity) or if the lactate concentration differences would have evened out (indicating increased flow of muscle lactate to the bloodstream as the consequence of an effect on the blood supply to the muscles). A further limitation is that we did not undertake a test-retest. However, to avoid the possible interaction of the factor time, we randomly assigned the experimental conditions ensuring that 50% of the subjects worked under one or other condition in each test session. In addition, as an inclusion criterion, participants had experience with the Wingate test.

## 5. Conclusions

Beetroot juice (containing 5.6 mmol NO_3_^−^) taken as a supplement had an ergogenic effect on maximum power production and a trend was observed for this to occur within the first 15 s of an inertial cycle ergometer Wingate test. The supplement also led to increased blood lactate concentrations post-exercise. We attribute these effects of BJ to specific improvements in power output and blood supply to type II motor units.

## Figures and Tables

**Table 1:** Characteristics of the 15 study participants.

Variable	M ± SD
Age (years)	21.46 ± 1.72
Height (cm)	1.78 ± 0.07
Weight (kg)	76.90 ± 8.67
BMI (kg/m^2^)	24.21 ± 1.72
Kilogram-force (Kp)	5.77 ± 0.64

BMI = body mass index; M ± SD = mean (±standard deviation).

**Table 2:** Metabolic variables and rating of perceived effort recorded in response to the Wingate test according to the experimental conditions (beetroot juice or placebo supplementation).

Variables	Placebo	CV (%)	BJ	CV (%)	%	*T*	*p*
Lactate-resting (mmol·L^−1^)	1.7 ± 0.45	26.6	2.0 ± 0.53	26.7	15.9	−2.051	0.059
Lactate-final (mmol·L^−1^) *	7.4 ± 2.84	38.0	13.6 ± 4.12	30.2	82.6	−5.337	0.000
RPE-muscular	17.33 ± 1.58	9.2	17.80 ± 1.14	6.4	2.7	−1.388	0.187
RPE-cardiovascular	16.53 ± 2.50	15.1	16.73 ± 1.70	10.2	1.2	−0.315	0.757
RPE-general	17.60 ± 1.88	10.7	17.86 ± 1.12	6.3	1.5	−0.459	0.653

BJ: beetroot juice; RPE = rating of perceived exertion; CV = coefficient of variation; * significant difference for placebo vs. BJ (*p* < 0.05). Data provided as the mean and standard deviation.

**Table 3:** Power variables recorded in the Wingate test in participants according to the experimental conditions (beetroot juice or placebo supplementation).

Variables	Placebo	CV (%)	BJ	CV (%)	%	*T*	*p*
Minimun power (W)	433.33 ± 99.39	22.9	442.61 ± 122.79	27.7	2.1	−0.264	0.796
Peak power (W) *	816.83 ± 136.97	16.8	865.69 ± 143.91	16.6	6.0	−2.357	0.034
Mean power (W)	613.98 ± 94.14	15.3	648.41 ± 104.79	16.2	5.6	−1.541	0.146
Time-to-peak power (s)	8.00 ± 1.46	18.3	7.33 ± 1.23	16.8	−8.4	2.092	0.055
Average power 0–5 s (W) *	530.34 ± 106.49	20.1	580.50 ± 109.87	18.9	9.5	−2.141	0.050
Average power 25–30 s (W)	462.46 ± 101.63	22.0	482.28 ± 112.73	23.4	4.3	−0.631	0.538
Fatigue index (%)	46.28 ± 12.01	25.9	48.65 ± 15.54	25.8	5.1	−0.701	0.495

BJ = beetroot juice; s = seconds; W = watts; CV = coefficient of variation; * significant difference for placebo vs. BJ (*p* < 0.05).
